# Artificial intelligence to predict the need for mechanical ventilation in cases of severe COVID-19

**DOI:** 10.1590/0100-3984.2022.0049

**Published:** 2023

**Authors:** Mariana Frizzo de Godoy, José Miguel Chatkin, Rosana Souza Rodrigues, Gabriele Carra Forte, Edson Marchiori, Nathan Gavenski, Rodrigo Coelho Barros, Bruno Hochhegger

**Affiliations:** 1 Pontifícia Universidade Católica do Rio Grande do Sul (PUCRS), Porto Alegre, RS, Brazil; 2 Universidade Federal do Rio de Janeiro (UFRJ), Rio de Janeiro, RJ, Brazil

**Keywords:** COVID-19, Tomography, X-ray computed, Artificial intelligence, COVID-19, Tomografia computadorizada, Inteligência artificial

## Abstract

**Objective:**

To determinate the accuracy of computed tomography (CT) imaging assessed by deep neural networks for predicting the need for mechanical ventilation (MV) in patients hospitalized with severe acute respiratory syndrome due to coronavirus disease 2019 (COVID-19).

**Materials and Methods:**

This was a retrospective cohort study carried out at two hospitals in Brazil. We included CT scans from patients who were hospitalized due to severe acute respiratory syndrome and had COVID-19 confirmed by reverse transcription-polymerase chain reaction (RT-PCR). The training set consisted of chest CT examinations from 823 patients with COVID-19, of whom 93 required MV during hospitalization. We developed an artificial intelligence (AI) model based on convolutional neural networks. The performance of the AI model was evaluated by calculating its accuracy, sensitivity, specificity, and area under the receiver operating characteristic (ROC) curve.

**Results:**

For predicting the need for MV, the AI model had a sensitivity of 0.417 and a specificity of 0.860. The corresponding area under the ROC curve for the test set was 0.68.

**Conclusion:**

The high specificity of our AI model makes it able to reliably predict which patients will and will not need invasive ventilation. That makes this approach ideal for identifying high-risk patients and predicting the minimum number of ventilators and critical care beds that will be required.

## Abstract (pt)

**Objetivo:**

Determinar a acurácia da tomografia computadorizada (TC), avaliada por redes neurais profundas, na ventilação mecânica, de pacientes hospitalizados por síndrome respiratória aguda grave por COVID-19.

**Materiais e Métodos:**

Trata-se de estudo de coorte retrospectivo, realizado em dois hospitais brasileiros. Foram incluídas TCs de pacientes hospitalizados por síndrome respiratória aguda grave e COVID-19 confirmada por RT-PCR. O treinamento consistiu em TC de tórax de 823 pacientes com COVID-19, dos quais 93 foram submetidos a ventilação mecânica na hospitalização. Nós desenvolvemos um modelo de inteligência artificial baseado em redes de convoluções neurais. A avaliação do desempenho do uso da inteligência artificial foi baseada no cálculo de acurácia, sensibilidade, especificidade e área sob a curva ROC.

**Resultados:**

A sensibilidade do modelo foi de 0,417 e a especificidade foi de 0,860. A área sob a curva ROC para o conjunto de teste foi de 0,68.

**Conclusão:**

Criamos um modelo de aprendizado de máquina com elevada especificidade, capaz de prever de forma confiável pacientes que não precisarão de ventilação mecânica. Isso significa que essa abordagem é ideal para prever com antecedência pacientes de alto risco e um número mínimo de equipamentos de ventilação e de leitos críticos.

## INTRODUCTION

Since coronavirus disease 2019 (COVID-19) was declared a pandemic by the World Health Organization, on March 11, 2020, various measures have been implemented worldwide in order to promote early diagnosis and containment of the disease^(1,2)^. In a study conducted in China^(3)^, the sensitivity of reverse transcription-polymerase chain reaction (RT-PCR) tests to identify infection with severe acute respiratory syndrome coronavirus 2 (SARS-CoV-2) was found to range from 37% to 71%. In another study, Fang et al.^(4)^ demonstrated that the sensitivity of chest computed tomography (CT) was significantly greater than was that of RT-PCR (98% vs. 71%; *p* < 0.001). Therefore, imaging came to be recognized as an important additional diagnostic tool during the pandemic.

According to the Fleischner Society, the indications for CT scans in patients with suspected COVID-19 include moderate to severe clinical features, regardless of laboratory test results, and worsening respiratory status in patients testing positive for infection with SARS-CoV-2^(5)^. In the early phase of COVID-19, CT typically shows bilateral ground-glass opacities, with a predominantly peripheral, subpleural distribution. Several days after the onset of symptoms, linear consolidations or areas with the reverse halo sign can appear, suggesting organizing pneumonia, which is associated with a poorer prognosis in older patients^(6)^.

In scenarios in which there is limited availability of radiologists, there can be a significant delay in providing chest CT reports, which are helpful to emergency physicians and clinicians engaged in the management of COVID-19. Therefore, it is important to develop a method to help physicians predict the severity of the viral disease, which we argue could be done through the use of artificial intelligence (AI).

Studies have shown that AI algorithms, particularly deep learning algorithms, perform remarkably well in classifying lung disease^(7,8,9)^. Deep learning is characterized as a subset of machine learning that is based on a neural network structure loosely inspired by the human brain. Convolutional neural networks (CNNs) currently represent the most prevalent deep learning architecture in medical imaging. These networks successively map image inputs to desired endpoints while learning increasingly reliable imaging features. Deep learning solutions have been proposed for the analysis of various imaging modalities, including CT^(8,10)^.

The aim of the present study was to determinate the accuracy of CT imaging assessed by deep neural networks in predicting the need for mechanical ventilation (MV) in patients hospitalized with SARS due to COVID-19.

## MATERIALS AND METHODS

This was a retrospective cohort study carried out at two tertiary hospitals in Brazil between April 1, 2020 and May 31, 2020. This study was approved by the institutional ethics committees of both hospitals.

We included CT scans from patients who were hospitalized due to SARS and had a diagnosis of COVID-19, as confirmed by RT-PCR. To identify SARS, we used the criteria established by the Brazilian National Ministry of Health^(11)^: flu symptoms with dyspnea; persistent chest tightness; oxygen saturation less than 95% on room air; or cyanosis of the lips or face. Patients for whom CT images were incomplete or unavailable were excluded from the study. The indications for MV included excessive respiratory effort, with evidence of muscle fatigue. The model predicted the risk for requiring MV within the first 72 h after admission.

### Patients and dataset

The initial dataset consisted of 947 CT scans of 833 consecutive inpatients. All of the cases were anonymized before inclusion in the study. Ten patients were excluded because a soft-tissue kernel was not identified in the CT dataset. The final sample comprised 937 CT scans, with a training set of 823 patients and a test set of 114 patients.

**Figure 1:**
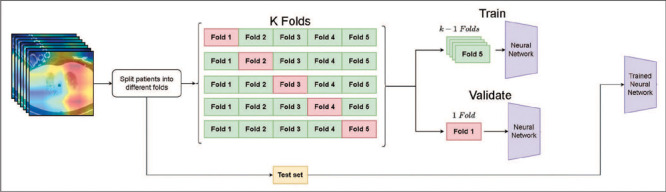
Randomization using the k-fold cross-validation procedure.

The training set consisted of chest CT examinations of 823 patients with COVID-19, of whom 93 required MV during hospitalization. We included only the first CT scan for each patient. The total number of slices in the training set was 189,290. We used k-fold cross-validation (k = 5) to compute the validation metrics (Figure 1). In this validation procedure, we trained the model five times, each time with different patients composing the training and validation sets, 80% of the data being used for training and 20% being used for validation. Each patient appeared in the validation fold once and in the training fold four times. The test set contained CT scans from 114 patients, of whom 67 required MV, with a total number of slices of 28,500. The model used in order to compute the metrics on the test set was trained over all the samples of the training set, rather than over samples from a particular fold.

### CT techniques

All chest CT examinations were performed in 64-slice scanners—LightSpeed VCT (GE Healthcare, Milwaukee, WI, USA) or Somatom Sensation 64 (Siemens AG, Forchheim, Germany)—and were acquired and reconstructed with soft-kernel reconstruction as axial images, with the following parameters: slice thickness, 1.25 mm; interslice gap, 1.25 mm; voltage, 120 kVp; and current, 200 mAs.

### AI model design

**Figure 2:**
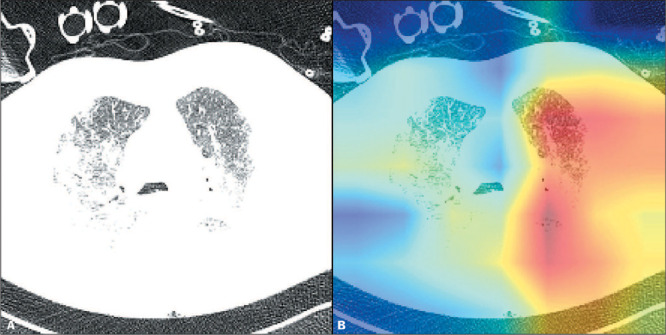
A: Axial unenhanced CT scan showing ground-glass opacities and consolidation in both lungs, findings typical of COVID-19. B: Heatmap of the same image, in which areas of red indicate activation of the algorithm related to prediction of the need for MV.

We developed an AI model based on CNNs, one of the most successful deep learning architectures to date^(12)^. In the past few years, CNNs achieved state-of-the-art results in several medical image analysis tasks^(13)^. By using a mathematical operation called convolution, which leads to local connections between neurons of adjacent layers and shared weights, CNNs exploit spatially-local correlations on the input data^(14)^, making them an excellent option for automated image analysis. Each convolutional layer has matrices of weights, also called filters or kernels, that are convolved with the inputs. Each resulting matrix is called a feature map, which summarizes the features of the input image in a lower-dimensional space (Figure 2). The filters within each convolutional layer are optimized during the training process to learn the best features to represent the desired output.

DenseNet-121^(15)^ is a CNN architecture with connectivity patterns that allow it to eliminate redundancies and thus has fewer parameters than do similar networks. CheXNet^(16)^, a CNN based on DenseNet-121, has been shown to achieve radiologist-level performance for detecting pneumonia on chest radiographs. In our proposed approach, each CT slice is an individual input during training and testing, which increases the number of input samples. To perform transfer learning from other computer vision tasks, we used a model pre-trained on the ImageNet dataset and then trained it on our dataset of CT slices for eight epochs. The input image size is 224 × 224 pixels. For image augmentation purposes, the slices may go through a random horizontal flip with a probability of 0.5. The network outputs a score between 0 and 1, representing the risk that the patient will require M V.

### Statistical analysis

Receiver operating characteristic curves, with their areas under the curve and 95% confidence intervals, were used in order to quantify the performance of the AI prediction models. Because the model evaluates the slices individually, we first computed the metrics for individual slices. Optimal thresholds (0.001) were obtained to describe the sensitivity, specificity, positive predictive value, negative predictive value, positive likelihood ratio, and negative likelihood ratio.

All statistical tests used were two-tailed, and a significance level of 5% was established. The analyses were performed with the Predictive Analytics Software package, version 18.0 (SPSS Inc., Chicago, IL, USA).

## RESULTS

**Figure 3:**
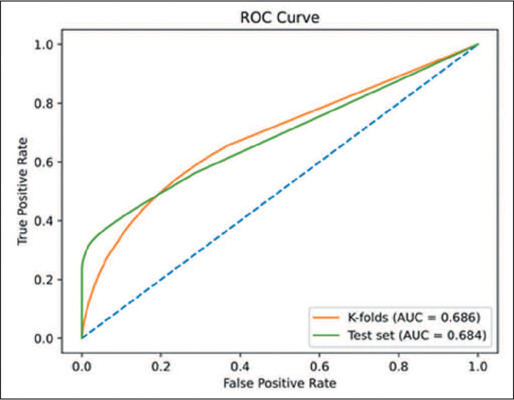
ROC curve computed by using either the k-fold cross-validation procedure or the test set.

As can be seen in Table 1, the AI model had an overall sensitivity of 0.417 and an overall specificity of 0.860. The receiver operating characteristic curve is shown in Figure 3. The corresponding area under the curve for the test set was 0.68. We prioritized specificity metrics, meaning that the model will rarely classify as “positive” patients who do not need MV.

**Table 1:** Individual slice metrics computed by using k-fold cross-validation and the test set.

Set	Acc	Sen	Spe	AUC	Total samples	Positive samples
K-folds	0.818	0.417	0.860	0.686	296,698	27,961
Test	0.560	0.391	0.921	0.684	166,716	113,703

Acc, accuracy; Sen, sensitivity; Spe, specificity; AUC, area under the curve.

## DISCUSSION

We have created a machine learning model with high specificity, capable of reliably predicting which patients will and will not require invasive ventilation. That makes this approach ideal for identifying high-risk patients and predicting the minimum number of ventilators and critical care beds that will be needed, which is extremely important during a pandemic, when intensive care units can be overwhelmed. Our study was conducted using only tomographic data. We see this as a strong point, because there is some difficulty in obtaining clinical data and complete medical records in a real-world setting, especially in low-and middle-income countries.

Previous studies have shown that the use of AI combining tomographic and clinical data has good accuracy for predicting critical evolution. Wang et al.^(17)^ employed an AI system to evaluate a sample of 1,051 patients with COVID-19, of whom 282 eventually required intensive care, required MV, or evolved to death. In that study, the AI concordance index for predicting critical illness was 0.8. The authors found that the AI system successfully stratified the patients into high-risk and low-risk groups with significantly different risks of progression. Another study, conducted at a single hospital in Mexico, with the objective of developing a multivariable prognostic model, evaluated clinical and chest CT data from 166 patients with COVID-19^(18)^. The authors found that a CT severity score had an area under the curve of 0.88 for predicting the need for M V, with a sensitivity of 65% and a specificity of 92%.

During the emerging COVID-19 pandemic, radiology departments faced a substantial increase in the number of requests for chest CT scans, together with the new demand for quantification of pulmonary opacities^(19)^. With overwhelming demands on medical resources, risk-based stratification of patients is essential. Given the large number of examinations in high case-load scenarios, an automated tool could facilitate and save critical time in the diagnosis and risk stratification of the disease. The AI model created for the present study could also facilitate hospital management and resource allocation.

Our study has some limitations. First, it used a retrospective design, with a likely selection bias. Second, a disadvantage of all deep learning methods is the lack of transparency and interpretability—e.g., it is currently quite difficult to determine what imaging features are being used in order to determine the output^(20)^.

In conclusion, our findings demonstrate that a deep learning model can reliably predict which patients will require invasive ventilation, with accuracy similar to that reported in the literature for other methods and without the need for clinical data assessment. Albeit promising, our AI model should be validated in multiple cohorts to evaluate its performance across populations and settings.
